# The Ubiquitin Ligase TRAF6 Negatively Regulates the JAK-STAT Signaling Pathway by Binding to STAT3 and Mediating Its Ubiquitination

**DOI:** 10.1371/journal.pone.0049567

**Published:** 2012-11-19

**Authors:** Juncheng Wei, Yanzhi Yuan, Chaozhi Jin, Hui Chen, Ling Leng, Fuchu He, Jian Wang

**Affiliations:** 1 State Key Laboratory of Proteomics, Beijing Proteome Research Center, Beijing Institute of Radiation Medicine, Beijing, China; 2 Institute of Basic Medical Science, Chinese Academy of Medical Science and Peking Union Medical College, Beijing, China; 3 Institutes of Biomedical Sciences, Fudan University, Shanghai, China; Semmelweis University, Hungary

## Abstract

STAT3 is a key transcription factor that mediates various cellular and organismal processes, such as cell growth, apoptosis, immune response and cancer. However, the molecular mechanisms of STAT3 regulation remain poorly understood. Here, we identified TRAF6 as a new STAT3 interactor. TRAF6 augmented the ubiquitination of STAT3 and deactivated its transcriptional activity induced by IFNα stimulation or overexpressed with JAK2. Both the RING domain and the TRAF-type zinc finger domain of TRAF6 were indispensable for STAT3 deactivation. Accordingly, TRAF6 also down-regulated the expression of two known STAT3 target genes, *CRP* and *ACT*. Therefore, we showed that TRAF6 is a new regulator of JAK/STAT signaling and provide a new mechanistic explanation for the crosstalk between the NF-κB and the JAK-STAT pathways.

## Introduction

Signal transducer and activator of transcription (STAT) proteins are identified as a family of latent cytoplasmic transcription factors that mediate cellular responses to cytokines, growth factors and other polypeptide ligands, including IL-6, interferons and epidermal growth factor [1]–[2]. Among the STAT proteins, STAT3 has been investigated more than others because of its various functions in diverse cellular processes [3]–[4]. The activation of STAT3 by phosphorylation is mediated by growth factor receptor tyrosine kinases and cytoplasmic kinases, such as cytokine receptor–associated Janus kinases (JAKs) and Src family kinases [5]–[6]. Then STAT3 is translocated from the cytoplasm to the nucleus, where it binds to specific DNA response elements and stimulates the expression of target genes [7].

Tumor necrosis factor receptor-associated factors (TRAFs) have been identified as signaling intermediates for TNFR superfamily members and are known to regulate various signaling pathways, including NF-κB, MAPK and Akt [8]–[11]. Unlike other TRAFs, TRAF6 participates in interleukin-1 receptor (IL-1R)/Toll-like receptor (TLR) superfamily signaling [12]–[13]. The RING finger domain of TRAF6 can function as a ubiquitin ligase that generates non-degradative K63-linked ubiquitin chains and mediates self-polyubiquitination [14]. Lysine 63-mediated ubiquitination is thought to add new functional properties to the modified protein, but does not promote proteasomal degradation. TRAF6 has been reported to directly associate with and ubiquitinate the transcription factor IRF7 [15]. TRAF6-deficient mice die at an early age, fail to active NF-κB and produce inflammatory cytokines [16]. However, it remains unknown whether TRAF6 is involved in the JAK-STAT signaling pathway and by what molecular mechanisms of this interaction occurs.

This study confirmed that TRAF6 functionally interacts with STAT3. Our results show, for the first time, that TRAF6 negatively regulates the activation of the JAK-STAT signaling pathway by binding to STAT3 and a novel mechanism exists in which an ubiquitin E3 ligase mediates the deactivation of STAT3.

## Materials and Methods

### Reagents, Antibodies and Plasmids

Monoclonal anti-Flag antibody and anti-Flag HRP was obtained from Sigma (St. Louis, MO). Protein A/G Plus-agarose, mouse polyclonal anti-TRAF6, rabbit polyclonal anti-STAT3, monoclonal anti-Myc (9E10), and anti-Myc (9E10) HRP antibodies were from Santa Cruz Biotechnology (Santa Cruz, CA). The expression plasmids for Flag-STAT3, HA-Ub, HA-Ub(K48R) and HA-Ub(K63R) were from Dr. Zhijie Chang’s lab (Tsinghua University, China). The GAS (containing IFN-γ-activating sequence), pACT (containing the promoter of α1–antichymotrypsin which has two STAT3 binding sites), m67 (a synthetic STAT3-responsive promoter), 4×IRF (containing four copies of the STAT-binding sequence from the interferon regulatory factor–1 gene) reporter genes were used as described [17], [18], [19], [20], [21]. TRAF6 was obtained by PCR using human liver cDNA library as a template and then inserted into pCMV-myc and pFlag-CMV vectors. The various fragments of TRAF6 were amplified by PCR, followed by subcloning into pFlag-CMV.

### Cell Culture and Transfections

Human embryonic kidney HEK293 cells (ATCC, Manassas, VA) were cultured in DMEM supplemented with 10% fetal bovine serum. Transfections were performed with Entranster-H (Engreen, China) according to the manufacturer's instructions.

### Immunoprecipitation and Immunoblotting

For general cell lysis and co-immunoprecipitation of STAT3 and TRAF6, HEK293 cells were transfected with the indicated vectors by Entranster-H. Cells were cultured for 1 day in DMEM medium and were lysed in lysis buffer (50 mM Tris–HCl (pH 7.5), 150 mM NaCl, 1% (v/v) Tween 20, 0.2% NP-40,10% glycerol) supplemented with protease inhibitor cocktail (Roche, Basel, Switzerland) and phosphatase inhibitors (10 mM NaF and 1 mM Na_3_VO_4_). Immunoprecipitations were performed using the appropriate antibodies and protein A/G-agarose (Santa Cruz, CA) at 4°C. Lysates and immunoprecipitates were incubated with the indicated primary antibodies and the appropriate secondary antibody, followed by detection with the SuperSignal West Pico Chemiluminescent Substrate (Thermo, IL).

### Luciferase Reporter Assays

HEK293 cells were transfected with 100 ng of the GAS, m67 or 4×IRF reporter genes of STAT3 plus 1 ng of the Renilla luciferase expression vector pRL-TK (Promega, WI), with or without the pCMV-Myc-TRAF6 vector. The cells were collected after treatment for 6 h with IFNα. The luciferase activity was measured with the Dual Luciferase Assay System as previously described [22]. All experiments were repeated at least three times.

### Quantitative Real Time RT-PCR

HEK293 cells were transfected with TRAF6. After transfection for 24 h, total RNA was isolated with TRIzol and the first strand cDNA was synthesized by reverse transcription PCR according to the manual (TOYOBO, China). Quantitative RT-PCR was run on a Bio-Rad IQ5 PCR machine with each PCR mixture containing 0.5 µl cDNA template and 10 nM primers in 25 µl of SYBR green reaction mix (TOYOBO, China). Expression values were normalized to those obtained with control *Gapdh*. The mRNA was measured using the primers listed as follows: *CRP*: forward: (5′-tcttggtcttgaccagcctctctc3’), reverse (5′-cataggaagtatccgactctttgg-3′); *Gapdh*: forward: (5′-aatcccatcaccatcttcca-3′), reverse (5′-tggactccacgacgtactca- 3′). Changes in gene expression levels were calculated by the 2^−ΔΔCt^ method.

## Results

### TRAF6 Interacts with STAT3 and Mediates the Ubiquitination of STAT3

**Figure 1:**
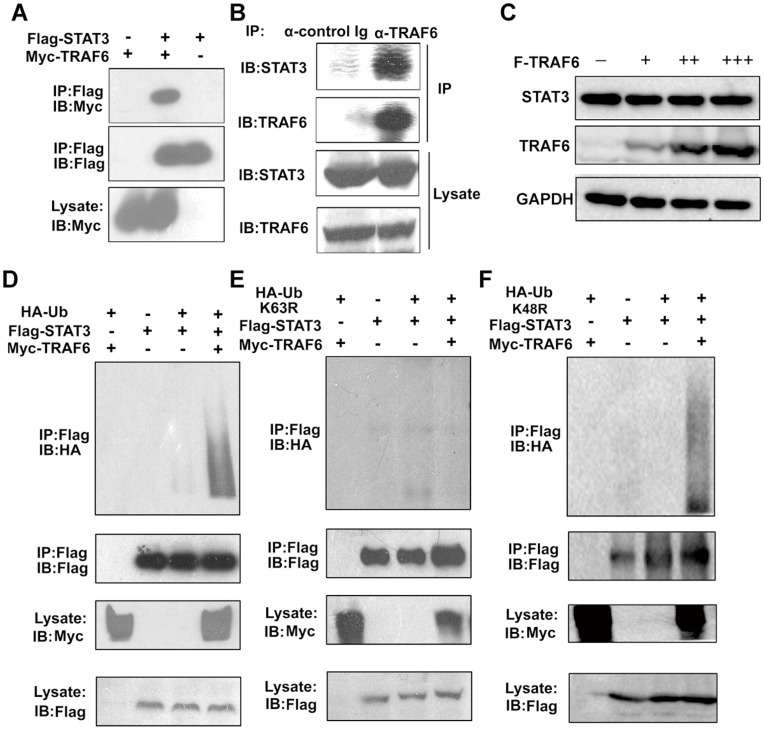
TRAF6 interacts with STAT3 and mediates the ubiquitination of STAT3. (A) TRAF6 interacts with STAT3 in mammalian cells. Myc-TRAF6 and Flag-STAT3 were co-transfected into HEK293 cells. After 24 h, the cell lysates were immunoprecipitated with anti-Flag antibody and subjected to Western blot with the anti-Flag or anti-Myc antibodies. (B) The cell lysates of HEK293 cells were immunoprecipitated with anti-TRAF6 antibody and subjected to Western blot with the anti-TRAF6 or anti-STAT3 antibodies. (C) Myc-TRAF6 was transfected into HEK293 cells in a dose-dependent manner. After 24 h, the cell lysates were probed with anti-Myc, anti-STAT3 and anti-GAPDH antibodies. (D, E and F) TRAF6 mediates the ubiquitination of STAT3. HA-Ub(WT) (D), HA-Ub(K63R) (E) or HA-Ub(K48R) (F) and Flag-STAT3 were co-expressed in HEK293 cells with Myc-TRAF6 or empty vectors. The cell lysates and immunoprecipitates were resolved by SDS-PAGE and immunoblotted with anti-HA or anti-Flag antibodies. The data are representative of at least three independent experiments.

We detected the interaction of TRAF6 and STAT3 by chance when used them as a negative control in a co-immunoprecipitation assay. To further test the interaction of TRAF6 and STAT3 in mammalian cells, Myc-TRAF6 and Flag-STAT3 proteins were co-expressed in HEK293 cells. The cell lysates were immunoprecipitated with an anti-Flag antibody, and the presence of associated proteins was determined by Western blot. The two proteins were shown to co-immunoprecipitate when an anti-Flag antibody was used to precipitate STAT3, as an anti-Myc antibody to detect TRAF6 in the immunoprecipitation. This result shows that STAT3 interacts with TRAF6 (Fig. 1A). To rule out the artificial results of the interaction of them, we further examined endogenous association of TRAF6 and STAT3 in HEK293 cells. As shown in Fig. 1B, endogenous TRAF6 physiologically binded to STAT3, but not with control IgG antibody. Furthermore, we found that the expression of TRAF6 did not affect the total amount of STAT3 (Fig. 1C).

TRAF6 is an E3 ubiquitin ligase and a scaffold protein that mediates K63 polyubiquitination of a few proteins, including NEMO, Akt and itself [8], [23]. To determine whether TRAF6 ubiquitinates STAT3, we examined the ubiquitination of STAT3 in the presence of overexpressed TRAF6. We observed the increased ubiquitination of STAT3 when co-transfected with TRAF6 (Fig. 1D). To confirm that TRAF6 mediates K63 linked ubiquitination of STAT3, the K63R and K48R mutants of ubiquitin were transfected with TRAF6 and STAT3. We found that TRAF6 failed to increase the ubiquitination of STAT3 when co-transfected with HA-Ub (K63R) (Fig. 1E, last lane). But TRAF6 still augmented the ubiquitination of STAT3 with HA-Ub (K48R) (Fig. 1F, last lane).

### TRAF6 Represses the Transcriptional Activity of STAT3

**Figure 2:**
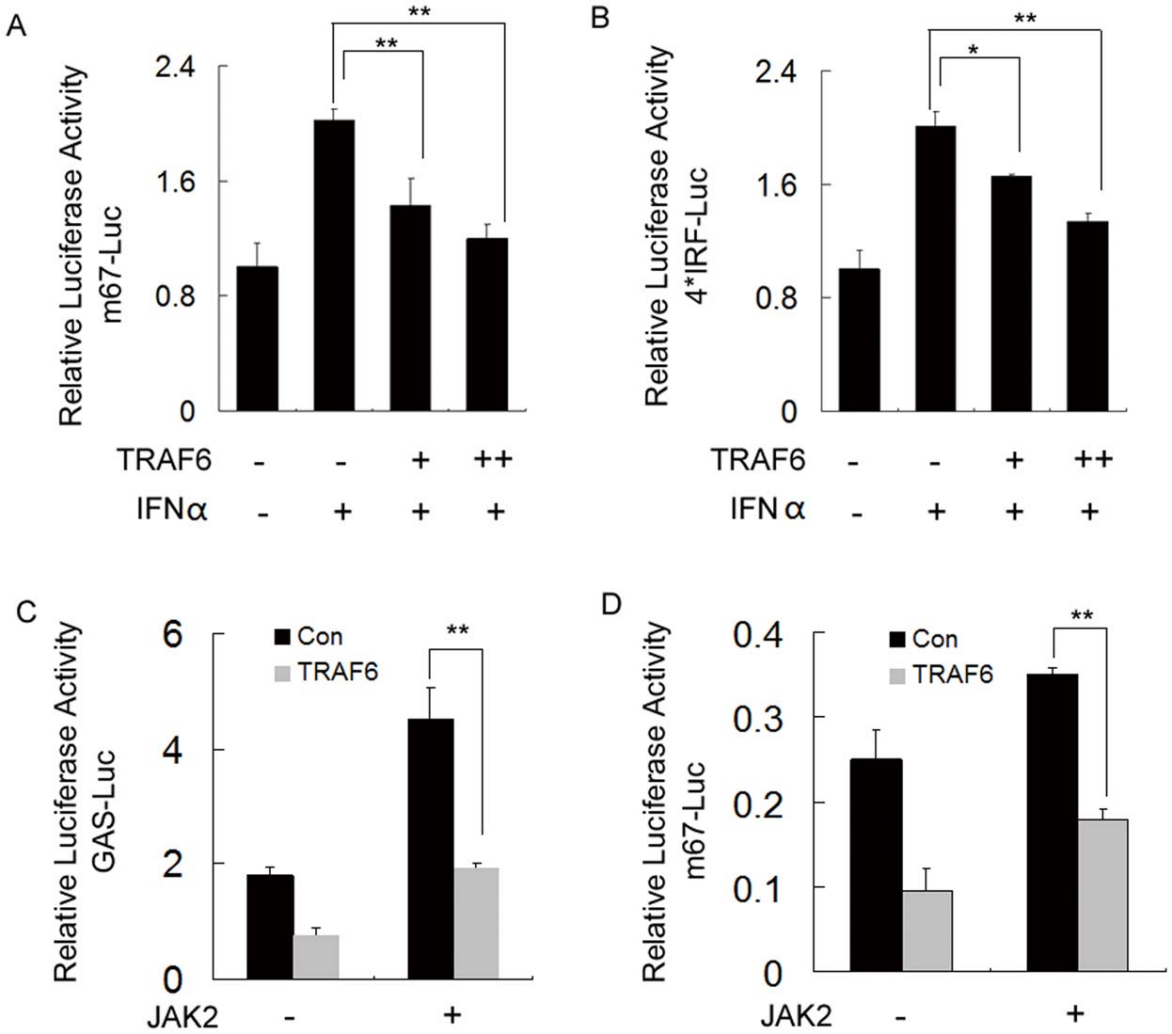
TRAF6 inhibits the transcriptional activity of STAT3. (A, B) TRAF6 represses the activity of STAT3 with IFNα stimulation. HEK293 cells were transiently transfected with the indicated combinations of m67 luciferase reporter or 4×IRF luciferase reporter, pRL-TK control, STAT3 and TRAF6. Then the cells were treated with IFNα (50 ng/ml) for 6 h before lysis. After 24 h, the cell lysates were collected for luciferase activity measurements. Data are presented as the means ± S.D. (n = 3). (C, D) TRAF6 inhibits the activity of STAT3 with the forced expression of JAK2. HEK293 cells were transiently transfected with GAS luciferase reporter or m67 luciferase reporter, TRAF6, STAT3 and JAK2. After 24 h, the cell lysates were collected for luciferase activity measurements. Data are presented as the means ± S.D. (n = 3).

To investigate the functional consequences of the interaction between TRAF6 and STAT3, we examined the effect of TRAF6 on the transcriptional activation of STAT3 using luciferase reporter genes that was under the control of the STAT3 response element. HEK293 cells were transfected with TRAF6 and m67-Luc (a synthetic STAT3-responsive promoter) [17]. As shown, overexpression of TRAF6 deactivated the transcriptional activity of STAT3 induced by the stimulation of the IFNα (Fig. 2A). To excluded the artificial effect that caused by m67-Luc, we performed the same experiments with another STAT3 responsive luciferase reporter, and the results showed that TRAF6 inhibits the 4×IRF reporter genes activity in a dose depend manner (Fig. 2B) [21]. Meanwhile, our results also demonstrated that TRAF6 repressed the JAK2, a STAT3 activator, induced GAS (IFNγ activating sequence)-Luc and m67-Luc signaling (Fig. 2C and 2D) [18], [20], [24].

### The RING Finger Domain and TRAF-type Zinc Finger Domain Play Key Roles in the Regulation of STAT3 Transcriptional Activity

**Figure 3:**
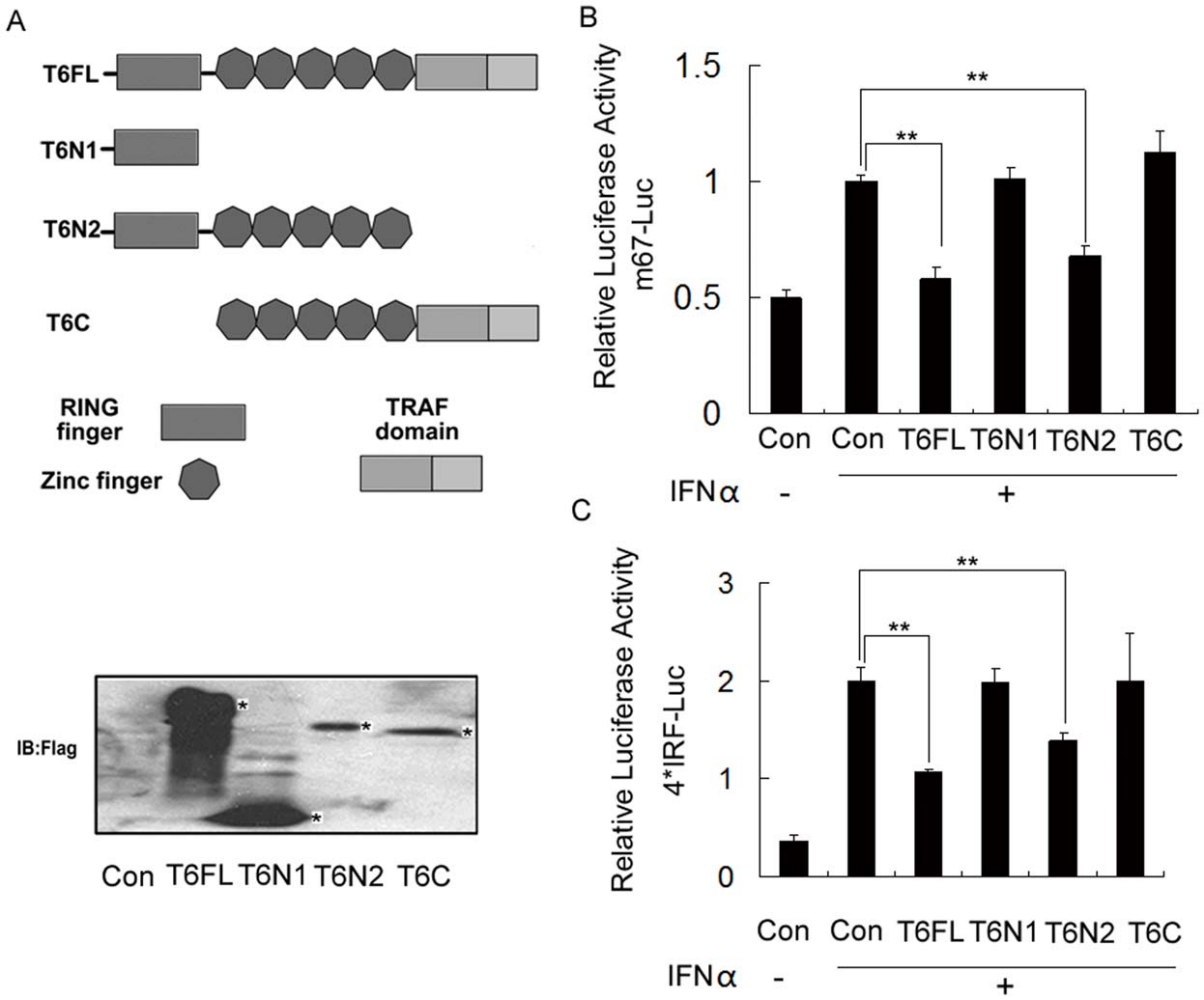
The RING finger domain and the TRAF-type zinc finger domain play key roles in the regulation of the transcriptional activity of STAT3. (A) A schematic illustration of TRAF6 domains. The asterisk indicates the expression of TRAF6 mutants that was detected by Western blot with anti-Flag antibody. (B and C) Reporter gene assays of STAT3. HEK293 cells were transiently transfected with various TRAF6 truncated mutants, m67 luciferase plasmids (B) or 4×IRF luciferase plasmids (C), STAT3 and pRL-TK plasmids. After 24 h, the cell lysates were collected for luciferase activity measurements. Data are presented as the means ± S.D. (n = 3).

TRAF6 consists of a RING finger domain followed by a series of putative zinc-finger motifs and a highly conserved TRAF domain [25]–[26] (Fig. 3A). The RING finger domain of TRAF6 functions as an ubiquitin ligase that cooperates with the Ubc13/Uev1A complex to mediate the polyubiquitination of many proteins [27]. To delineate the domain of TRAF6 that regulates the activity of STAT3, HEK293 cells were transfected with m67-Luc, full length TRAF6 or the deletion mutants. Our study showed that TRAF6 truncation (T6C) lacking the RING domain was unable to deactivate the transcriptional activity of STAT3 (Fig. 3B, lane 6). The TRAF6 truncation (T6N1) only containing the RING domain was also not able to inhibit the transcription of STAT3 (Fig. 3B, lane 4). However, the TRAF6 truncation (T6N2) containing the RING and the TRAF-type zinc finger domains did effectively deactivate STAT3 (Fig. 3B, lane 5). Accordingly, we demonstrated that T6N2 but not other deletion mutants of TRAF6 could inhibit the 4×IRF reporter activity (Fig. 3C). These results suggest that the RING and the TRAF-type zinc finger domains of TRAF6 cooperate with each other to negatively control the activation of STAT3.

### TRAF6 Down-regulates the Expression of the STAT3 Target Genes

**Figure 4:**
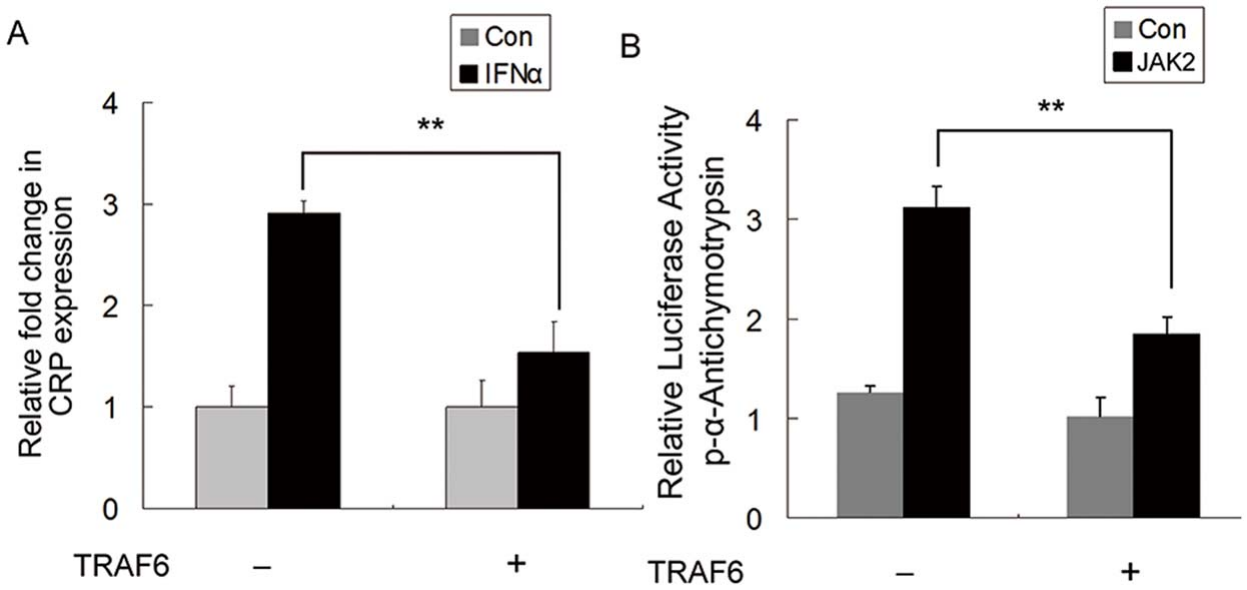
TRAF6 negatively regulates the expression of the STAT3 target genes. (A) TRAF6 inhibited the expression of the STAT3 target gene, *CRP*. The HEK293 cells were transfected with the indicated amounts of TRAF6. After 24 h, the HEK293 cells were stimulated with IFNα for 15 min and the mRNA levels of *CRP* were examined by real-time PCR. The expression values were normalized to the expression of *GAPDH*. Data are presented as the means ± S.D. (n = 3). (B) TRAF6 inhibited the expression of *ACT*. HEK293 cells were transiently transfected with the indicated combinations of pACT luciferase reporter, pRL-TK control TRAF6, STAT3 and JAK2. After 24 h, the cell lysates were collected for luciferase activity measurements. Data are presented as the means ± S.D. (n = 3). These results are representative of three independent experiments. (**P<0.01 compared with control samples which are not transfected with TRAF6, Student's t test).

The activity of STAT3 is critical for the expression of many genes, including α-Antichymotrypsin (ACT) and C-reactive protein (CRP), which are important for immune response and inflammation [19], [28]. To further test the subsequent biological effects of TRAF6 on the transcription of downstream target genes of STAT3, we analyzed the expression levels of CRP by real-time PCR in HEK293 cells. We found that TRAF6 inhibits the expression of CRP induced by IFNα (Fig. 4A). To confirm the effect of TRAF6 on the STAT3 target gene, HEK293 cells were transfected with pACT-Luc (a luciferase reporter plasmid containing the promoter of ACT), TRAF6 and JAK2. The results showed that TRAF6 also inhibits the expression of α-Antichymotrypsin (Fig. 4B).

## Discussion

Both of the NF-κB and the JAK-STAT signaling pathways have a central role in the immune response, but the cross-talk between the two signaling pathways is not very clear [1] [11]. The NF-κB transcription factor p65 is shown to physically bind to STAT3 and inhibit the transcription function of STAT3. In contrast, p50 cooperates with STAT3 to enhance its activity [29]. Previously, we demonstrate that the inhibitor of NF-κB signaling pathway, IκBζ, interacts with STAT3 and negatively regulates the transcription activity of STAT3 [22]. But the molecular mechanisms of cytokines, such as IL-1 and IFN, simultaneously activate the NF-κB and the STAT3 signaling pathways remain elusive.

STAT3 is the major signal transducer downstream of cytokines and oncogenes, such as IFNs, Src, Ras, and ErbB2 [1] [6] [30]–[31], and is involved in many distinct functions, including the activation of acute-phase response, stimulation of proliferation, induction of terminal differentiation and growth arrest. The post-translational modification of STAT3 is critical for its functions, such as phosphorylation, acetylation and methylation [32], [33]. Tyrosine phosphorylation by JAK or Src kinases is required for dimerization, nuclear translocation and DNA binding of STAT3 under cytokine stimulation. STAT3 dimerization is also regulated by acetylation at lysine 685 stimulated with ligand of CD44 [32]. Ubiquitination also plays a key role in regulating of the degradation of STAT3. STAT3 is ubiquitinated and degraded by the 26S proteasome with treatment of TMF/ARA160 [34]. However, it remains unknown whether STAT3 could be directly ubiquitinated without leading to its degradation.

TRAF6 functions as a ubiquitin E3 ligase that mediates the addition of non-degradative K63-linked ubiquitin chains to several key proteins in the NF-κB pathway that activate IκB kinase (IKK) in response to proinflammatory cytokines [11] [16]. Recently, TRAF6 has been shown to directly ubiquitinate Akt kinase, which is important for its membrane localization and phosphorylation [8]. In our results, we show for the first time that the ubiquitin E3 ligase TRAF6 binds to STAT3 *in vivo* using coimmunoprecipitation assays in mammalian cells. As an E3 ligase, TRAF6 is also reported to bind to and ubiquitinate IRF7, a transcription factor that is responsible for virus or TLR ligand-induced expression of type I interferon [15]. In this context, our data demonstrate that the transcription factor STAT3 is an additional ubiquitinated target of TRAF6. This result suggests that TRAF6 might attach lysine 63-linked polyubiquitin chains to STAT3 to regulate its activation.

It has been showed that a core TT-AA motif with a 4 to 6 spacing displaying general STAT binding [35]. In this study, we chose a STAT3 reporter gene (m67) to measure the effect of TRAF6 on the activation of STAT3 [20] [24]. To reduce the interference of other Stat proteins, we overexpressed STAT3 with the luciferase reporters. The results showed that TRAF6 inhibits the transcriptional activity of STAT3 in a dose dependent manner. To further confirm the negative regulation of STAT3 by TRAF6, we performed the luciferase experiments with 4×IRF and GAS reporter genes [21]. It should be noted that these reporter genes are not only responsive to STAT3. We overexpressed Stat3 with the luciferase reporters to reduce the interference of other STAT proteins, which has been generally used to measure the transcriptional activity of Stat3 [17] [20]–[21]. The results further validated that TRAF6 negatively regulates the STAT3 activity in a dose dependent manner. The TRAF domain at the C-terminus of TRAF6 interacts with the TRAF-C domain to form the cap for association with receptors and adaptor proteins, the coiled-coil motif of TRAF6 is identified as the stalk for trimerization, and the RING domain comprises the core of the ubiquitin ligase catalytic domain [26]. To determine the regions that mediate the deactivation of STAT3 by TRAF6, the truncated mutants of TRAF6 were transfected to HEK293 cells with STAT3 luciferase reporter genes. The results showed that the RING domain and the zinc-finger motifs are critical for the regulation of the transcriptional activity of STAT3 (Figure 3). Interestingly, the truncation mutant containing the RING domain and the zinc-finger motifs is also able to enhance the activity of AP-1 and NF-κB transcription factors.

STAT3 mediates the expression of a variety of genes and has a critical role in many cellular processes, such as T cells proliferation, immune response and inflammation [36]. The STAT3 target genes have been implicated in a number of cancers, including liver, breast, and lung carcinomas, as well as skin disease and autoimmunity [7] [37]–[39]. We also examined the effect of TRAF6 on the induction of STAT3-dependent target genes, including C-reactive protein and α-Antichymotrypsin. We found that both of these genes were down-regulated by overexpression of TRAF6.

The direct ubiquitination of transcriptional factors plays a key role in the activition of several signaling pathways [40], [41]. As an ubiquitin E3 ligase, TRAF6 binds to the transcription factors IRF7 and Smad2/3 [15], [42]. This study provides evidences of the functional interaction of TRAF6 and STAT3. The detailed molecular mechanisms by which TRAF6 deactivates the JAK-STAT pathway should be investigated further, as well as its biological roles in antiviral response, immune and inflammatory response and cancer.
